# Potential Health-Promoting Effects of Two Candidate Probiotics Isolated from Infant Feces Using an Immune-Based Screening Strategy

**DOI:** 10.3390/nu14173651

**Published:** 2022-09-03

**Authors:** Huijing Liang, Xiaolei Ze, Silu Wang, Yimei Wang, Chenrui Peng, Ruyue Cheng, Fengling Jiang, Simou Wu, Ruikun He, Fang He, Xuguang Zhang, Xi Shen

**Affiliations:** 1 Department of Nutrition and Food Hygiene, West China School of Public Health and West China Fourth Hospital, Sichuan University, Chengdu 610041, China; 2 BYHEALTH Institute of Nutrition & Health, Guangzhou 510663, China; 3 Department of Infectious Disease Prevention, Sichuan Tianfu New Area Public Health Center, Chengdu 610213, China

**Keywords:** probiotics, intestinal microbiota, immunity, short-chain fatty acids, screening

## Abstract

Commensal microorganisms in the human gut are a good source of candidate probiotics, particularly those with immunomodulatory effects that may improve health outcomes by regulating interactions between the gut microbiome and distal organs. Previously, we used an immune-based screening strategy to select two potential probiotic strains from infant feces in China, *Bifidobacterium breve* 207-1 (207-1) and *Lacticaseibacillus paracasei* 207-27 (207-27). In this study, the in vitro immunological effects and potential in vivo general health benefits of these two strains were evaluated using *Lacticaseibacillus rhamnosus* GG (LGG) as the control. The results showed that 207-1 and 207-27 significantly and differentially modulated the cytokine profiles of primary splenic cells, while did not induce abnormal systemic immune responses in healthy mice. They also modulated the gut microbiota composition in a strain-dependent manner, thus decreasing Gram-negative bacteria and increasing health-promoting taxa and short-chain fatty acid levels, particularly butyric acid. Conclusively, 207-1 and 207-27 shaped a robust gut environment in healthy mice in a strain-specific manner. Their potential immunomodulatory effects and other elite properties will be further explored using animal models of disease and subsequent clinical trials. This immune-based screening strategy is promising in efficiently and economically identifying elite candidate probiotics.

## 1. Introduction

Probiotics are described as “live microorganisms that, when administered in adequate amounts, confer a health benefit on the host” [1]. The field of probiotics has advanced considerably in recent years as the role of human microbiota in health and disease has been better understood [2,3]. Numerous studies have been devoted to discovering new candidate probiotics with better characteristics and health-promoting properties. Among the many potential sources, commensal microorganisms in the human gut are often considered as a good source of candidate probiotics as they are more likely to be safe for humans, have easier intestinal colonization, and have a more specific application [4]. However, there remains a lack of standardized protocols for the isolation, identification, screening, and functional evaluation of potential probiotic strains, particularly those that are time-saving, low-cost, and repeatable.

According to the guidelines published by the Food and Agriculture Organization of the United Nations/World Health Organization (FAO/WHO), a candidate probiotic should fulfill several basic criteria including resistance to gastric acidity and bile acid, adherence to mucus or epithelial cells, antagonistic to pathogens, and safe [5]. Furthermore, probiotics are considered to confer certain general health benefits such as producing short-chain fatty acids (SCFAs), supporting a healthy digestive tract, a healthy gut microbiota, and a healthy immune system [1]. Among these health benefits, the immunological effects of a candidate probiotic are considered more strain-specific [1]. Accumulating evidence supports that the immune system is a key communication pathway between the gut microbiome and distal organs as it is involved in the regulation of metabolic homeostasis, behavior patterns, psychopathology, and cardiovasology. Hence, probiotics with immunomodulatory effects, particularly anti-inflammatory effects, are expected to confer more health outcomes by regulating the interaction with distal organs [6,7,8,9]. Therefore, the immunological effects can be used as the front and primary criterion for screening strains, thus accelerating the narrowing of the range of candidate probiotics.

Based on the above evidence, we established an immune-based screening strategy to efficiently and economically screen for candidate probiotics from infant feces. As shown in Figure S1, after the conventional isolation and identification processes [10,11], bacterial strains were tested for the ability to resist gastric and bile acid, adhere to mucus or epithelial cells, and antagonize pathogens. Once the stress-resistant phenotype was confirmed, several murine macrophage cell lines were used to investigate the immunological properties of the strains. Murine macrophage cell lines were selected as they are easy to culture and relatively low cost, which helped to quickly narrow the range of candidate strains. After these processes, two strains with excellent immunological properties were selected, namely *Bifidobacterium breve* 207-1 (207-1) and *Lacticaseibacillus paracasei* 207-27 (207-27). These were found to activate RAW264.7 macrophages when co-cultured for 5 min (or less) and modulated the cytokine profiles, inducing the production of the anti-inflammatory cytokine interleukin (IL)-10 [11,12]. The mechanism may be related to the activation of p44/42 MAPK (ERK), P38, and nuclear factor κB (NF-κB) phosphorylation [12].

Based on the previous findings, the present study aimed to further verify the immunological effects of 207-1 and 207-27 by using another in vitro immune cell model and evaluated the potential health benefits in vivo using *Lacticaseibacillus rhamnosus* GG (LGG) as the control strain. The immunomodulatory effects of these two strains on primary murine splenic cells were evaluated and compared with previous findings on a single cell line. The regulatory effects on systematic immunity, intestinal microbiota, and microbial metabolites were then investigated in healthy mice. This study is one of the important steps of the immune-based screening strategy for selecting candidate probiotics and provides fundamental data for subsequent animal and clinical studies.

## 2. Materials and Methods

### 2.1. Mice

A total of 36 six-week-old BALB/c male mice were purchased from Beijing Vital River Laboratory Animal Technology Co. Ltd. (Beijing, China). They were housed in individually ventilated cages with ad libitum access to water and food under specific pathogen-free conditions. The ambient temperature (23 ± 1 °C), humidity (50–70%), and light conditions (12/12-h light/dark cycle) were strictly controlled. After adaptive feeding for 7 days, mice were used for the isolation of primary splenic cells or the administration of bacterial strains. The Experimental Animal Management Committee of Sichuan Government approved the animal experimental facility and experimental protocols used in this study (approval number: SYXK2018-011).

### 2.2. Preparation of Bacterial Cultures and Suspensions

LGG was kindly supplied by Chr. Hansen Holding A/S (Hørsholm, Denmark). 207-1 and 207-27 were originally isolated from healthy infant feces in China [10,11] and they are now deposited at the Guangdong Microbial Culture Collection Center (GDMCC) under the Budapest Treaty, with deposit codes GDMCC 60962 and GDMCC 60960, respectively. 207-27 is also named *Lacticaseibacillus* *paracasei* LPB27 in its commercialized product. LGG and 207-27 were cultured on de Man, Rogosa, and Sharpe agar (Land Bridge, Beijing, China) and 207-1 were cultured on transoligosaccharide propionate (Eiken, Tochigi, Japan). Bacterial strains were cultured at 37 °C for 48 h under anaerobic conditions and passaged three times before use. Then, cultures were collected and washed twice with sterile saline. The concentrations of bacterial suspensions were determined by the colony-forming unit (CFU)-absorbance standard curves of each created bacterial strain.

### 2.3. Isolation and Treatment of Primary Murine Splenic Cells

After adaptive feeding, a total of four mice were sacrificed by cervical dislocation. The spleens of mice were aseptically removed and cut into small pieces in RPMI-1640 medium containing 300 mg/L L-glutamine (Gibco, Carlsbad, CA, USA), 10% heat-inactivated fetal bovine serum (Gibco), 100-U/mL penicillin, and 100-μg/mL streptomycin (Merck Millipore, Burlington, MA, USA). They were then separated mechanically into single cells using 70-μm cell strainers (Falcon, Oxnard, CA, USA) and washed twice with the medium above-mentioned. The cells were resuspended and seeded onto 24-well plates at 5 × 10^5^ cells/well. Next, the cells were co-cultured with LGG, 207-1, or 207-27 at 1 × 10^7^ CFU/well at 37 °C in 5% CO_2_ for 1, 3, or 24 h. RPMI-1640 medium was used as a negative control.

### 2.4. Animal Experimentation

After adaptive feeding, a total of 32 mice were randomly divided into the control (Ctrl; *n* = 6), LGG (*n* = 6), 207-1 (*n* = 10), and 207-27 (*n* = 10) groups and gavaged with 0.2-mL sterile saline, LGG suspension, 207-1 suspension, and 207-27 suspension each day for 4 weeks, respectively. Bacterial suspensions were administered at a concentration of 5 × 10^9^ CFU/mL.

At the end of the experiment, fresh stool pellets from mice were collected and frozen at −80 °C. All of the mice were then weighed and sacrificed by cervical dislocation. Blood samples were collected and centrifuged to isolate sera. The thymus, spleen, lung, and liver of mice were isolated and calculated for organ indices. The cecal content of mice was also collected and frozen at −80 °C.

### 2.5. Enzyme-Linked Immunosorbent Assay (ELISA)

After co-culture for 24 h, the levels of IL-6, IL-10, IL-12, and tumor necrosis factor (TNF)-α in the supernatant of primary splenic cells were assessed using ELISA kits (R&D systems, Minneapolis, MN, USA) in accordance with the manufacturer’s protocol. Additionally, the levels of these cytokines in the serum of mice were measured via the Luminex assay (R&D systems, Minneapolis, MN, USA) using a Luminex 200™ multiplexing instrument (Merck Millipore).

### 2.6. RNA Isolation and RT-qPCR

According to the manufacturer’s instructions, the Cell Total RNA Isolation Kit (Foregene, Chengdu, China) and Animal Total RNA Isolation Kit (Foregene) were used to isolate the total RNA from the primary splenic cells and spleen samples of the mice, respectively. The iScript cDNA Synthesis Kit (Bio-Rad, Hercules, CA, USA) was used to reverse transcribe the RNA into cDNA with a C1000 Touch Thermal Cycler (Bio-Rad). The synthesis conditions were as follows: priming at 25 °C for 5 min, reverse transcript at 46 °C for 20 min, inactivate reverse transcriptase at 94 °C for 1 min, and finally, held at 4 °C. Next, real-time quantitative PCR was performed as we mentioned previously [11] using the CFX96 touch system (Bio-Rad). A total of 10 μL reaction mix consisted of 1 μL cDNA, 0.3 μL forward primer, 0.3 μL reverse primer, 3.4 μL double-distilled water, and 5 μL SYBR Green Supermix (Bio-Rad). The thermal cycling protocol was as follows: polymerase activation and DNA denaturation at 98 °C for 30 s, followed by 39 cycles of denaturation at 98 °C for 5 s, and annealing and extension for 5 s (IL-6: 55 °C; IL-10: 57.7 °C; IL-12: 59.5 °C; TNF-α: 59.5 °C; β-actin: 64.5 °C). The melt curve analysis was undertaken and each sample was assayed twice for quality control. Relative mRNA levels were normalized against β-actin. The primer sequences of qPCR are shown in Table S1.

Primer sequences (5′–3′) were as follows: IL-6 (F: GTCACAGAAGGAGTGGCTA, R: AGAGAACAACATAAGTCAGATACC), IL-10 (F: GACCAGCTGGACAACATACT, R: GAGGGTCTTCAGCTTCTCAC), IL-12 (F: CTCTGTCTGCAGAGAAGGTC, R: GCTGGTGCTGTAGTTCTCAT), TNF-α (F: CTCTTCAAGGGACAAGGCTG, R: CGGACTCCGCAAAGTCTAAG), and β-actin (F: GTGGGCCGCTCTAGGCACCAA, R: CTCTTTGATGTCACGCACGATTTC). All primers were synthesized by Sangon Biotech (Shanghai, China).

### 2.7. Western Blotting

Western blotting was performed as previously described [11]. Briefly, after co-culture with the tested bacterial strains for 1 and 3 h, primary murine splenic cells were collected, washed twice, and lysed thoroughly with cell lysis buffer (Beyotime, Shanghai, China) on ice. The cell lysates were then treated with sodium dodecyl sulfate–polyacrylamide gel electrophoresis sample loading buffer (Beyotime), boiled for 5 min, separated by sodium dodecyl sulfate–polyacrylamide gel electrophoresis (120 V, 75 min), and transferred onto 0.45-μm polyvinylidene difluoride membranes (200 mA, 50 min; Merck Millipore). After blocking in 5% skim milk for 70 min, the membranes were blotted with anti-phosphorylated nuclear factor κB (p-NF-κB), anti-phosphorylated P38 (p-P38), anti-phosphorylated p44/42 MAPK (p-ERK), anti-NF-κB, anti-P38, anti-ERK, and anti-β-actin (Cell Signaling Technology, Beverly, MA, USA) overnight at 4 °C. Then, blots were incubated with secondary horse radish peroxidase-conjugated anti-rabbit or anti-mouse IgG antibody (Absin, Shanghai, China) for 50 min. The targeted proteins were visualized using the ECL luminescence reagent (Absin) in the ChemiDoc XRS+ (Bio-Rad).

### 2.8. Cecal SCFA Detection

The levels of acetic acid, propionic acid, and butyric acid in the cecal contents of mice were detected by a gas chromatography Agilent 7890B (Agilent; Santa Clara, CA, USA). Briefly, cecal contents (100 mg each) were homogenized with 100 μL phosphoric acid (15%; Sinopharm, Shanghai, China), 100 μL isohexanoic acid (50 μg/mL; Sigma-Aldrich, St Louis, MO, USA), and 400 μL ether (Sinopharm) for 1 min. After centrifugation, the supernatants were collected and detected via gas chromatography. The SCFA levels in the samples were calculated by standard curves made using acetic acid, propionic acid, and butyric acid standards (Sigma-Aldrich).

### 2.9. Fecal DNA Extraction and 16S rRNA Sequencing

The TIANamp Stool DNA Kit (Tiangen, Beijing, China) was used to extract the total DNA from the feces of mice, as per the manufacturer’s instructions. Using previously reported methods [13], fecal DNA was amplified by 16S rRNA genes (V3–V4) and sequenced on an Illumina MiSeq Instrument (Illumina, San Diego, CA, USA). The sequences of universal primers (5′–3′) were as follows: V3-338F (ACTCCTACGGGAGGCAGCAG), V4-806R (GGACTACHVGGGTATCTAAT).

### 2.10. Bioinformatic Analysis

Using previously reported methods [13], effective sequencing data were filtered and the chimeras were removed to obtain high-quality sequences for subsequent analysis. Effective tags were then clustered into operational taxonomic units (OTUs) with a 97% cut-off similarity. The SINTAX algorithm [14] implemented by USEARCH based on the RDP database were used to assign OTUs taxonomically. The USEARCH cluster_agg command was used for multiple sequence alignment and phylogenetic tree construction. The α- and β-diversity were assessed in Phyloseq v.1.30.0 using the OTU abundance table and phylogenetic tree [15]. The relative abundance of a microbe was calculated as the read count normalized by the total reads in that sample, while microbes with relative abundance <1% in all samples were classified as “others”. The linear discriminant analysis effect size (LEfSe) algorithm [16] was used to identify differentially abundant biomarkers in different treatments.

### 2.11. Statistical Analyses

The 16S rRNA sequencing data analysis was performed using R v.3.6.3 (R Core Team, Vienna, Austria). Other statistical analyses were performed using SPSS v.25.0 (SPSS Inc., Chicago, IL, USA). Data are represented as mean ± the standard error of the mean (SEM). The Kruskal–Wallis test or one-way ANOVA followed by the Bonferroni test was used for multiple comparisons. A probability (*p*) value < 0.05 indicated statistical significance.

## 3. Results

### 3.1. Immunomodulatory Effects of the Tested Strains on Primary Murine Splenic Cells

**Figure 1:**
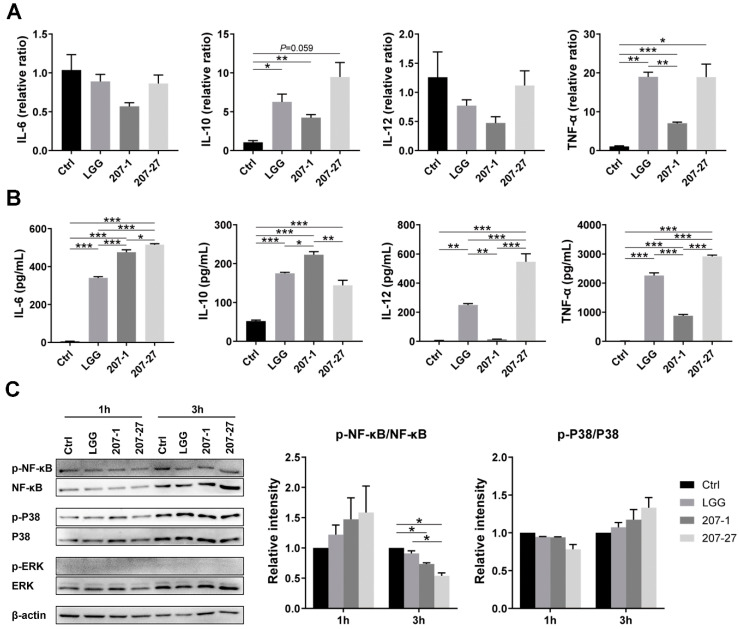
The immunomodulatory effects of the tested strains on the primary murine splenic cells. (**A**) Cytokine mRNA levels in the primary murine splenic cells after co-culture with the tested strains for 24 h (*n* = 4). (**B**) Cytokine levels in the supernatant of primary murine splenic cells after co-culture with the tested strains for 24 h (*n* = 4). (**C**) The representative protein blots and phosphorylation levels of NF-κB, P38, and ERK in the primary murine splenic cells after co-culture with the tested strains for 1 and 3 h (*n* = 3). Data are presented as mean ± SEM. * *p* < 0.05, ** *p* < 0.01, *** *p* < 0.001.

The cytokine profiles of primary murine splenic cells were determined via RT-qPCR and ELISA after being co-cultured with the tested bacterial strains for 24 h (Figure 1A,B). Compared with the Ctrl group, 207-1 was found to significantly induce IL-10 and TNF-α mRNA expressions as well as the secretion of IL-6, IL-10, and TNF-α. Consistent with our previous study using murine macrophage cell lines [11,12], 207-1 stimulated strong IL-10 production but little IL-12 production. However, 207-27 and LGG induced similar cytokine profiles that differed from 207-1. Compared with the Ctrl group, 207-27 and LGG both induced the IL-10 and TNF-α mRNA expressions and the secretion of IL-6, IL-10, IL-12, and TNF-α, while compared with the 207-1 group, they induced significantly more secretion of IL-12 and TNF-α, but less IL-10.

To determine whether the tested bacterial strains could activate P38/ERK/NF-κB signaling as found in the murine macrophage cell lines [12], we then analyzed the protein profiles in primary murine splenic cells at 1 and 3 h (Figure 1C). Compared with the Ctrl group, none of the tested strains was found to induce the phosphorylation of P38, ERK, or NF-κB (*p* > 0.05); 207-1 and 207-27 attenuated the phosphorylation of NF-κB (*p* < 0.05 for both) when co-cultured with primary murine splenic cells for 3 h.

### 3.2. Bodyweight and Organ Indices of Mice

**Figure 2:**
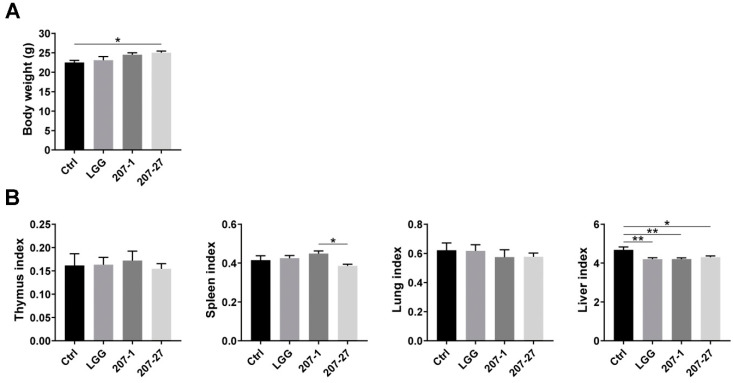
The body weight and organ indices of mice in each group (*n* = 6–10). (**A**) The body weight of mice at the end of the experiment. (**B**) The organ indices (g/100 g·bw) of mice were calculated. Data are presented as mean ± SEM. * *p* < 0.05, ** *p* < 0.01.

At the end of the experiment, the health status of mice in each group was found to be normal, without weight loss, illness, or death. As shown in Figure 2A, mice administered 207-27 showed higher body weights than those in the Ctrl group (*p* < 0.05). No statistically significant difference was noted in the thymus, spleen, or lung index between the Ctrl and bacterial strain-treated groups (*p* < 0.05), while LGG, 207-1, and 207-27 treatments were determined to decrease the liver index (*p* < 0.01, *p* < 0.01, and *p* < 0.05, respectively; Figure 2B).

### 3.3. Splenic and Serum Cytokine Levels in Mice

**Figure 3:**
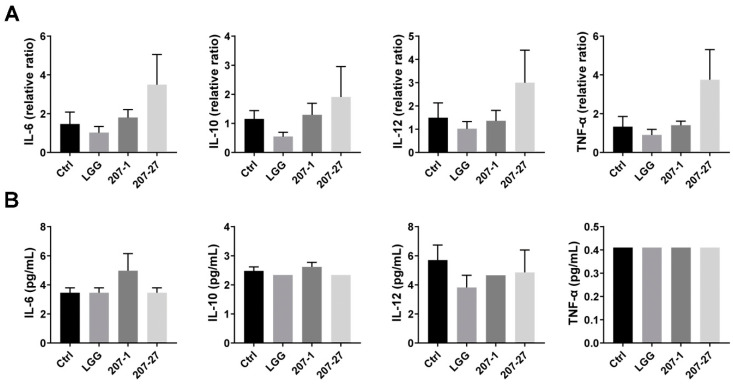
The splenic and serum cytokine levels of the mice in each group (*n* = 6–10). (**A**) Splenic cytokine mRNA expression levels. (**B**) Serum cytokine levels. Data are presented as mean ± SEM. Some data are missing the SEM because only one sample was detected, or the detected value was equal to the detection limit.

As shown in Figure 3A, none of the tested bacterial strains stimulated significant changes in the cytokine mRNA expression of healthy mice, which differed from the results of the in vitro experiments. Meanwhile, 207-27 treatment seemed to increase the mRNA levels of IL-6, IL-10, IL-12, and TNF-α, but the differences were deemed not statistically significant (*p* > 0.05 for all). Furthermore, we assessed the changes in the serum cytokine profiles of mice (Figure 3B). Similarly, no statistically significant differences were observed among the groups.

### 3.4. Changes in Fecal Microbial Diversity

**Figure 4:**
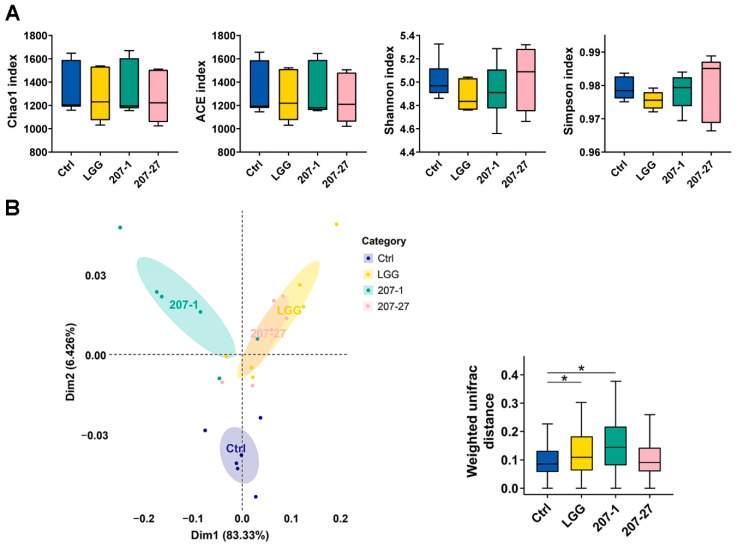
The α- and β-diversity analysis of the fecal microbiota of mice in each group (*n* = 6). (**A**) Box plot of the α-diversity index of the fecal microbiota. (**B**) PCoA plot of the fecal microbiota; the community differentiation in each group was measured by the weighted Unifrac algorithm. * *p* < 0.05.

At the end of the experiment, changes in the diversity of the fecal microbiota communities were analyzed using 16S rRNA sequencing. As shown in Figure 4A, there was no statistically significant difference in either community richness (indicated by Chao1 and ACE indices) or community evenness (indicated by Shannon and Simpson indices) between all treatments. Then, principal coordinate analysis (PCoA) based on weighted UniFrac distance was performed to reveal differences in the fecal microbial construction among communities regarding the evolutionary information and OTU abundance. As shown in Figure 4B, the fecal microbiota of mice in the LGG and 207-1 groups were classified into different clusters compared with that in the Ctrl group, which showed significant alterations in microbial communities due to the LGG and 207-1 treatments (*p* < 0.05 for both). Fecal microbiota in the LGG and 207-27 groups could not be clearly separated by PCoA, which indicates that mice in these groups shared a core set of intestinal bacteria.

### 3.5. Changes in Fecal Microbial Composition

To elucidate the specific changes in the fecal microbiota composition of mice after different treatments, the relative abundance of fecal microbiota at the phylum and genus levels were then analyzed and statistical analyses were performed. Notably, due to the database we used, the taxonomic note on the *Lactobacillus* genus still adopted the traditional classification method rather than the latest classification method proposed by Zheng et al. in 2020 [17].

**Figure 5:**
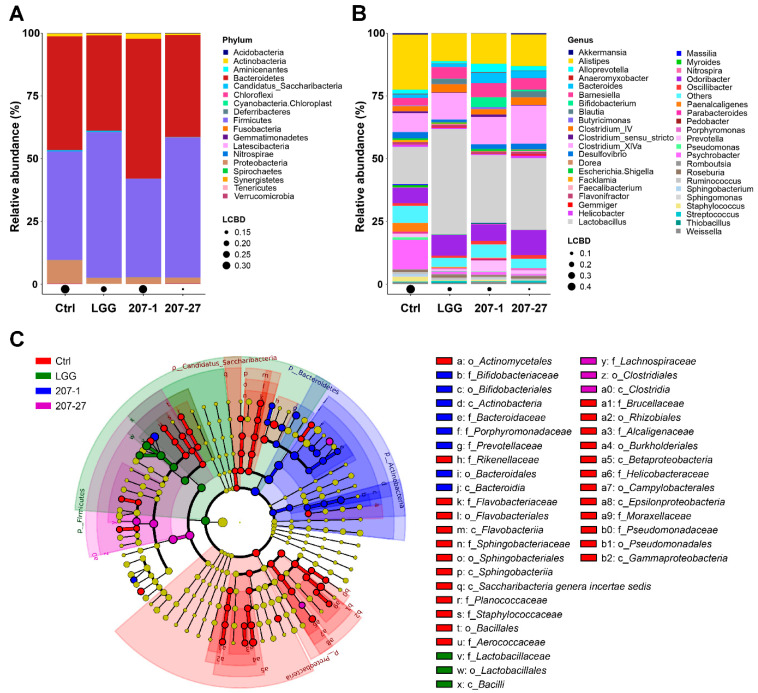
The fecal microbial composition of mice in each group (*n* = 6). (**A**) The fecal microbiota composition at the phylum level. (**B**) The fecal microbiota composition at the genus level. (**C**) The cladogram produced following LEfSe analysis. The meaning of the letter before various taxa: p—phylum; c—class; o—order; f—family; g—genus; s—species.

At the phylum level (Figure 5A), the fecal microbiota of mice was found to be dominated by *Bacteroidetes*, *Firmicutes*, *Proteobacteria*, and *Actinobacteria*. Mice administered 207-1 had a higher relative abundance of *Actinobacteria* than in the Ctrl group (2.07% vs. 1.12%; *p* < 0.05). Furthermore, the 207-27 and LGG groups shared a similar fecal microbial composition profile, which is consistent with the PCoA results. The relative abundances of *Firmicutes* in the 207-27 and LGG groups (55.78% and 58.01%) were higher than in the Ctrl group (43.22%; *p* < 0.01 for both). Additionally, the tested bacterial strains significantly decreased the abundance of *Proteobacteria* in the fecal microbiota of mice (*p* < 0.05 for all).

The major genera with the mean relative abundance are shown in Figure 5B. Comparing the 207-1 group with the Ctrl group, the relative abundance of *Lactobacillus* (27.03% vs. 14.93%), *Barnesiella* (5.63% vs. 3.03%), *Bacteroides* (4.03% vs. 1.35%), *Prevotella* (4.34% vs. 1.41%), and *Bifidobacterium* (3.95% vs. 0.13%) showed a significant improvement, whereas that of *Alistipes* (12.16% vs. 21.93%) and *Psychrobacter* (1.04% vs. 11.76%) exhibited a significant decrease. Comparing the 207-27 group with the Ctrl group, *Lactobacillus* (28.56% vs. 14.93%), *Clostridium_XlVa* (15.25% vs. 7.54%), *Odoribacter* (9.99% vs. 6.13%), *Barnesiella* (4.64% vs. 3.03%), *Bacteroides* (2.85% vs. 1.35%), and *Bifidobacterium* (0.62% vs. 0.13%) showed a prominent increase, whereas *Alistipes* (12.60% vs. 21.93%) and *Psychrobacter* (0.64% vs. 11.76%) showed a significant decrease. Comparing the LGG group with the Ctrl group, *Lactobacillus* (42.08% vs. 14.93%), *Odoribacter* (8.23% vs. 6.13%), and *Barnesiella* (4.74% vs. 3.03%) showed a significant increase, whereas *Alistipes* (11.09% vs. 21.93%), *Psychrobacter* (0.99% vs. 11.76%), and *Desulfovibrio* (1.01% vs. 2.47%) showed a decrease.

As per the LefSe analysis and the cladogram (Figure 5C), the specific phylotypes (biomarkers) that were responsive to 207-1 treatment were *Bacteroidaceae*, *Prevotellaceae*, *Porphyromonadaceae* (all from the class *Bacteroidia* and order *Bacteroidales*), and *Bifidobacteriaceae* (from the class *Actinobacteria* and order *Bifidobacteriales*). *Clostridiaceae* (from the class *Clostridia* and order *Clostridiales*) was also identified as a biomarker responsive to 207-27 treatment. Furthermore, the LGG group was characterized by *Lactobacillaceae* (from the class *Bacilli* and order *Lactobacillales*).

### 3.6. SCFA Levels Were Increased by Altered Gut Microbiota

**Figure 6:**
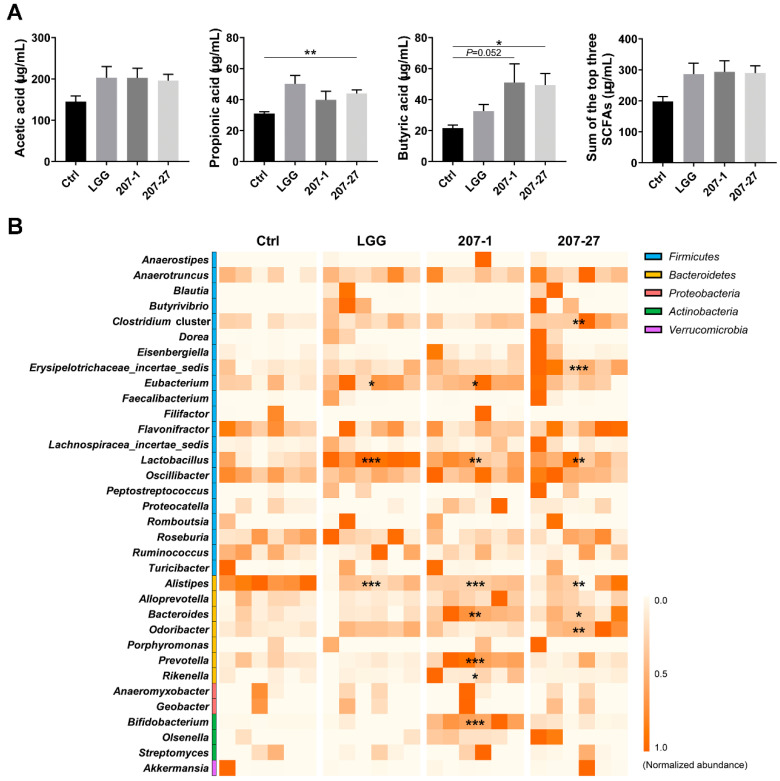
The tested strains increased the SCFA levels in the cecum and the relative abundance of the SCFA-producing genera in the fecal microbiota of mice (*n* = 6). (**A**) The SCFA levels in the cecal contents. Data are presented as the mean ± SEM. * *p* < 0.05, ** *p* < 0.01. (**B**) The heat map illustrating the effect of different treatments on the SCFA-producing genera identified by 16S rRNA sequencing (*n* = 6). Orange signifies that the genus was highly abundant, and white signifies that the genus was present in low abundance or absent. Compared with the Ctrl group, * *p* < 0.05, ** *p* < 0.01, *** *p* < 0.001.

The levels of the three major SCFAs in the cecal contents of mice were analyzed (Figure 6A). As per our findings, 207-27 treatment significantly increased the propionic and butyric acid levels in the cecal contents (*p* < 0.01 and *p* < 0.05, respectively). The 207-1 treatment also enhanced the butyric acid levels (*p* = 0.052). Although the differences were not statistically significant, all of the tested strains increased the total amount of the three major SCFAs (nearly 100 μg/mL).

The impact of the tested strains on the SCFA-producing genera [18,19,20] were further summarized, with relative abundances higher than 1% (Figure 6B). Compared with the Ctrl group, the relative abundances of six SCFA-producing genera were significantly increased by 207-1, while five were increased by 207-27 and two by LGG (*p* < 0.05 for all). These results suggest that the tested strains increased the cecal SCFA levels by regulating the intestinal microbiota and increasing the relative abundances of the SCFA-producing genera.

## 4. Discussion

The immunomodulatory properties of the selected probiotic strains can bring about a number of health benefits not only to the immune system, and these properties are highly strain-specific [6,7,8,9]. For example, the probiotic strain *Lacticaseibacillus gasseri* TMC0356 was found to reduce adipocyte hypertrophy and alleviate metabolic syndrome in rats by exerting its potent immunoregulatory effect [21,22,23]. Thus, in the early stage of strain screening, it is worth noting the immunological properties of potential probiotic strains.

Numerous studies have revealed that the immunomodulatory effects of probiotics can act directly by activating macrophages or natural killer cells, regulating the production of cytokines or immunoglobulins, or indirectly by enhancing the intestinal barrier function, modifying the gut microbiota composition or regulating intestinal metabolites [24,25]. In our selection criteria, we first used the murine macrophage cell lines to evaluate the immunological properties of potential strains in activating the macrophages and modulating cytokine profiles, as these cells are easily cultured and rapidly proliferated. However, a single cell line cannot reflect all of the immunological characteristics of the strains. Therefore, in this study, the immunological effects of 207-1 and 207-27 were further verified on murine primary splenic cells, which may be closer to the immune response in vivo.

Consistent with the results of macrophage cell lines [11,12], both strains were found to significantly activate primary splenic immune cells to secrete cytokines, and 207-1 characteristically induced an anti-inflammatory cytokine profile (high IL-10 and low IL-12 and TNF-α). IL-10 is known to be produced by monocytes, macrophages, B cells, T cells, and dendritic cells. It plays a crucial role in inhibiting pro-inflammatory cytokines (such as IL-1, IL-12, and TNF-α), chemokines, and chemokine receptors, thus alleviating intestinal inflammation and maintaining immune homeostasis [9]. Based on the above findings, further investigation on the potential role of 207-1 in treating chronic inflammatory diseases such as inflammatory bowel disease (IBD), irritable bowel syndrome, allergy, type 2 diabetes (T2D), and obesity is of critical significance [26]. In this study, 207-1 and 207-27 did not activate P38/ERK/NF-κB signaling, as previously discovered using a macrophage cell line [12]. This may be due to the complexity of the action of bacterial strains on the primary splenic cells, involving various immune cell types, receptors, and effector signals. These results also indicate that more than one in vitro model is required to evaluate the immunological properties of bacterial strains. In addition, the penicillin added to the culture medium may have a slight effect on the viability of bacterial strains, although the amount of penicillin is low and its pharmacological effect is to inhibit the proliferation of bacteria rather than directly kill the existing bacteria. The effect of antibiotics in the cell culture medium on the immunomodulatory function of probiotics needs further study.

Then, we preliminarily investigated the in vivo outcomes of 207-1 and 207-27 in healthy adult mice and discovered no statistically significant differences in systemic cytokine profiles among the groups. Similarly, Mohr et al. systematically reviewed 18 randomized controlled trials and discovered a limited effect of probiotic supplementation on circulating the immune and inflammatory markers in healthy adults [27]. This is most likely because healthy individuals have a well-functioning immune system, thus making it difficult to demonstrate how probiotics alter the immune response and inflammation. When studying the consistency of the in vitro immunomodulatory properties of probiotics in vivo, researchers have tended to choose a pathological condition such as experimental colitis [28]. Hence, the immunomodulatory effects of our strains should be further examined in terms of specific health pathologies (e.g., IBD, allergy, obesity, and psychological stress) or life stages (e.g., old age). Alternatively, this may partly reflect the in vivo safety of our tested strains, as they did not elicit excessive immune stimulation, according to the FAO/WHO guidelines [5].

Gut microbiota comprises about 70% of the entire microbiota population and has a significant influence on several aspects of human health including immune, metabolic, and neurobehavioral traits [2]. Targeting the gut microbiome with probiotics has been shown to benefit human health against pathological changes and diseases [3]. In this study, PCoA based on a weighted Unifrac algorithm revealed that the administration of 207-1 and LGG significantly altered the microbial communities in the feces of mice, indicating their strong ability to regulate the intestinal microbiota. Furthermore, 207-27 and LGG treatments induced highly similar changes in fecal microbial abundance and community diversity, indicating a similarity in their regulation of intestinal microbiota, although changes in the 207-27 group were not statistically significant. Additionally, the 207-27 and LGG groups shared a similar fecal microbial composition profile, particularly at the phylum level. These results indicate that the regulation of intestinal microbiota by probiotics may thus be genus-specific.

In this study, 207-1 and 207-27 significantly decreased the relative abundance of *Proteobacteria* by approximately 75%, which is a Gram-negative taxon containing a series of pathogens and is apparently related to inflammatory diseases including IBD, T2D, obesity, and HIV infection [8,29]. The abundance of *Psychrobacter* (from the phylum *Proteobacteria*) was also reduced by the tested strains; however, the health effects of this genus remain unclear. Additionally, 207-1 and 207-27 significantly decreased the relative abundance of *Alistipes* by nearly 50%, a relatively new genus of the *Bacteroidetes* phylum, which was recently found to be relevant in dysbiosis and disease [30]. As a butyrate producer [18], *Alistipes* seems to be protective against some inflammatory diseases [31,32]; however, it contrastingly has been shown to play a pathogenic role in mental health and glycolipid metabolism. Increased levels of *Alistipes* were also found to be associated with depression [33], anxiety [34], T2D [35], and high-fat diet-induced obesity [36,37,38]. Collectively, these changes in the microbial composition indicate the possible effects of 207-1 and 207-27 in the competitive inhibition of pathogens. Their potential to ameliorate mental and metabolic health deserves further exploration.

Notably, the administration of *B. breve* 207-1 significantly increased the relative abundance of *Lactobacillus* (traditional classification) by approximately 80% in the feces of mice, whereas *L. paracasei* 207-27 markedly increased the relative abundance of *Bifidobacterium* by nearly 400%. Possible contamination could be excluded as *L. rhamnosus* GG intervention did not increase fecal *Bifidobacterium* abundance. These results suggest that interventions of 207-1 and 207-27 increased not only the abundance of their respective genera, but also *Lactobacillus* (traditional classification) and *Bifidobacterium*, respectively, whose health benefits have been widely recognized [39,40]. Additionally, 207-1 increased the relative abundance of *Prevotella*, 207-27 increased *Clostridium_XlVa* and *Odoribacter*, and they both increased *Barnesiella* and *Bacteroides*. Among these genera, *Barnesiella* spp. have been considered as beneficial taxa as they enable the clearance of antibiotic-resistant *Enterococcus* [41] and positively impact the host metabolism [42,43]. Furthermore, other genera are all SCFA producers and would likely benefit the host [18,20].

The impact of our strains on the SCFA-producing genera and SCFA levels were further investigated. The results showed that 207-1 and 207-27 improved the abundances of some and different SCFA-producing genera, leading to an increase in the SCFA levels, particularly butyric acid. SCFAs are well-studied microbial metabolites and are considered to be important mediators in the communication between the gut microorganism and the immune system, helping to maintain immune homeostasis [20]. Recent studies have revealed that signals produced by SCFAs are not only transmitted in immune cells through free fatty acid receptors, but also directly to adipose tissue, affecting energy metabolism, glucose homeostasis, adipogenesis, and lipolysis and the browning of adipose tissue. Moreover, SCFAs can regulate the gut–brain and gut–liver axes, thus modulating the entire body [44]. To sum up, 207-1 and 207-27 contributed to building a robust gut environment including the intestinal microbiome and microbial metabolites in a strain-dependent manner. These promising results provide a solid foundation for further research on the elite properties of these two strains such as anti-pathogenicity, anti-inflammatory, anti-allergy, anti-obesity, anti-depression, and anti-anxiety.

The traditional selection criteria for potential probiotic strains mainly focus on stress-resistant phenotypes that guarantee their survival through the gastrointestinal tract and their subsequent existence. However, not all strains screened by these criteria offer biological benefits. In this study, an immune-based screening strategy were used to efficiently and economically screen out two candidate probiotics that support a healthy gut environment and a healthy immune system. This strategy is also being used to screen for potential probiotic strains from breast milk, which is another ideal source of probiotics, and have found good repeatability. Our strategy may provide a reference for researchers in selecting and evaluating new candidate probiotics.

Conclusively, two candidate probiotic strains isolated from infant feces using an immune-based screening strategy, namely, 207-1 and 207-27, imparted general benefits to healthy mice including shaping a robust gut microbiota, increasing the SCFA production, and possibly modulating immune responses in a strain-dependent manner. Their potential immunomodulatory effects and other elite properties will be further explored using animal models of disease and subsequent clinical trials. The immune-based screening strategy is a promising method to efficiently and economically screen out elite candidate probiotics.

## Data Availability

The 16S rRNA sequencing data reported in this study were submitted to the Sequence Read Archive (SRA) under the BioProject ID PRJNA822858 in NCBI (https://www.ncbi.nlm.nih.gov/sra/PRJNA822858, accessed on 31 August 2022).

## Supplementary Materials

The following supporting information can be downloaded at: https://www.mdpi.com/article/10.3390/nu14173651/s1, Table S1: qPCR primer sequences, Figure S1: Flow chart of the immune-based screening strategy for selecting candidate probiotics.
