# Supplementary material for: Potential Health-Promoting Effects of Two Candidate Probiotics Isolated from Infant Feces Using an Immune-Based Screening Strategy
Source: Nutrients. 2022 Sep 3;14(17):3651. doi: 10.3390/nu14173651 (PMC9459954; doi:10.3390/nu14173651)
Supplement: Supplementary file 1 [file nutrients-14-03651-s001.zip › nutrients-1865462-supplementary.pdf]

Supplementary Materials

Table S1. qPCR primer sequences.

Primer	Sequence (5'-3')
β-actin-F	GTGGGCCGCTCTAGGCACCAA
β-actin-R	CTCTTTGATGTCACGCACGATTTC
IL-6-F	GTCACAGAAGGAGTGGCTA
IL-6-R	AGAGAACAACATAAGTCAGATACC
IL-10-F	GACCAGCTGGACAACATACT
IL-10-R	GAGGGTCTTCAGCTTCTCAC
IL-12-F	CTCTGTCTGCAGAGAAGGTC
IL-12-R	GCTGGTGCTGTAGTTCTCAT
TNF-α-F	CTCTTCAAGGGACAAGGCTG
TNF-α-R	CGGACTCCGCAAAGTCTAAG

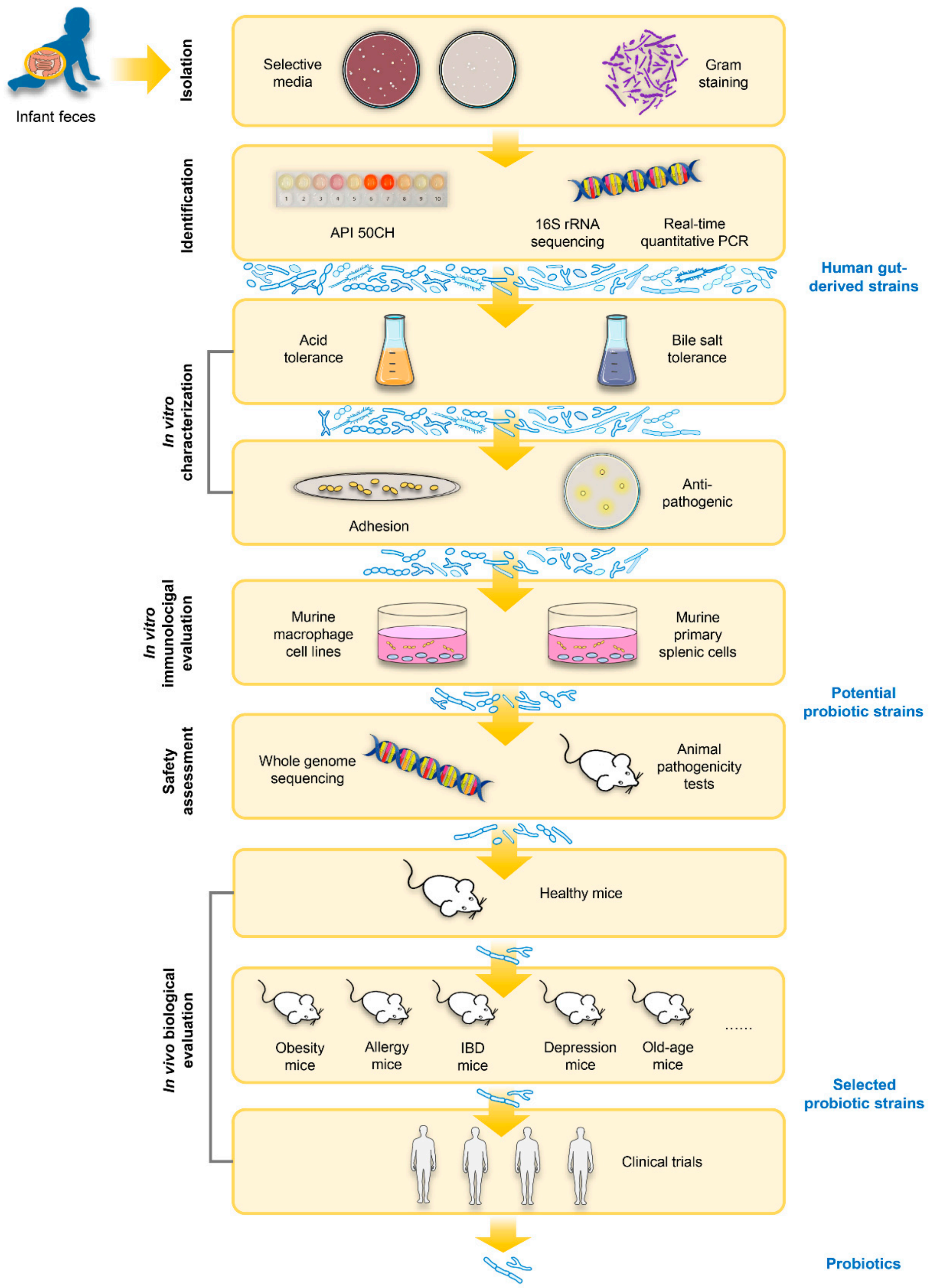

Figure S1. Flow chart of the immune-based screening strategy for selecting candidate probiotics. In our previous studies, candidate probiotics were screened from the feces of healthy infants in Chengdu, China. Selective media were used to isolate bifidobacteria and lactobacilli. API 50CH biochemical test, 16S rRNA sequencing, and real-time quantitative PCR (qPCR) with specific primers were used in identification [10,11]. Then, *in vitro* assays were performed to evaluate the ability of these isolates to resist gastric and bile acid, adhere to mucus or epithelial cells, and antagonize pathogens. Isolates with stress-resistant phenotype were co-cultured with murine macrophage cell

lines to detect their immunomodulatory potential [11,12]. The safety of potential probiotic strains was then assessed using whole-genome sequencing and animal pathogenicity tests. This study further verified the immunological effects of these strains on murine primary splenic cells and evaluated their health benefits in healthy mice. The elite properties of the candidate probiotics will be further explored using animal models of disease and subsequent clinical trials.
